# Differential Expression of mRNAs in the Brain Tissues of Patients with Alzheimer's Disease Based on GEO Expression Profile and Its Clinical Significance

**DOI:** 10.1155/2019/8179145

**Published:** 2019-02-26

**Authors:** Guowei Ma, Mingyan Liu, Ke Du, Xin Zhong, Shiqiang Gong, Linchi Jiao, Minjie Wei

**Affiliations:** ^1^School of Pharmacy, Department of Pharmacology, China Medical University, Shenyang, Liaoning, China; ^2^Liaoning Key Laboratory of Molecular Targeted Anti-Tumor Drug Development and Evaluation, Shenyang, Liaoning, China

## Abstract

**Background:**

Early diagnosis of Alzheimer's disease (AD) is an urgent point for AD prevention and treatment. The biomarkers of AD still remain indefinite. Based on the bioinformatics analysis of mRNA differential expressions in the brain tissues and the peripheral blood samples of Alzheimer's disease (AD) patients, we investigated the target mRNAs that could be used as an AD biomarker and developed a new effective, practical clinical examination program.

**Methods:**

We compared the AD peripheral blood mononuclear cells (PBMCs) expression dataset (GEO accession GSE4226 and GSE18309) with AD brain tissue expression datasets (GEO accessions GSE1297 and GSE5281) from GEO in the present study. The GEO gene database was used to download the appropriate gene expression profiles to analyze the differential mRNA expressions between brain tissue and blood of AD patients and normal elderly. The Venn diagram was used to screen out the differential expression of mRNAs between the brain tissue and blood. The protein-protein interaction network map (PPI) was used to view the correlation between the possible genes. GO (gene ontology) and KEGG (Kyoto Gene and Genomic Encyclopedia) were used for gene enrichment analysis to determine the major affected genes and the function or pathway.

**Results:**

Bioinformatics analysis revealed that there were differentially expressed genes in peripheral blood and hippocampus of AD patients. There were 4958 differential mRNAs in GSE18309, 577 differential mRNAs in GSE4226 in AD PBMCs sample, 7464 differential mRNAs in GSE5281, and 317 differential mRNAs in GSE129 in AD brain tissues, when comparing between AD patients and healthy elderly. Two mRNAs of RAB7A and ITGB1 coexpressed in hippocampus and peripheral blood were screened. Furthermore, functions of differential genes were enriched by the PPI network map, GO, and KEGG analysis, and finally the chemotaxis, adhesion, and inflammatory reactions were found out, respectively.

**Conclusions:**

ITGB1 and RAB7A mRNA expressions were both changed in hippocampus and PBMCs, highly suggested being used as an AD biomarker with AD. Also, according to the results of this analysis, it is indicated that we can test the blood routine of the elderly for 2-3 years at a frequency of 6 months or one year. When a patient continuously detects the inflammatory manifestations, it is indicated as a potentially high-risk AD patient for AD prevention.

## 1. Introduction

AD is a kind of dementia that was first discovered and named by the German psychiatrist and neuropathologist Alzheimer Alois in 1906 [1]. AD is a chronic neurodegenerative disease from mild-to-severe symptoms with the main clinical manifestations such as memory loss, cognitive dysfunction, behavioral abnormalities, and social disorders [2]. In 2015, there were 46.8 million AD patients worldwide, and it is expected to reach 74.7 million in 2030 and more than 131.5 million in 2050. The global costs of AD care in 2015 totaled up to 818 billion dollars. In just three years, this amount will increase to 1 trillion dollars, and in 2030 it will reach 2 trillion dollars [3]. If the early detection and intervention are available and effective, the onset will be delayed for 5-10 years, which can exceedingly reduce the family burden and medical system pressure [4]. Therefore, the early diagnosis of Alzheimer's disease is extremely important.

Many hypotheses were proposed in the pathogenesis of AD. As we know, the main pathogenesis of AD was the abnormal deposition of amyloid *β*-protein (A*β*), hyperphosphorylation of Tau protein, oxidative stress, mitochondrial cascade, inflammatory response, and barrier to insulin signaling pathways. However, AD was a complex disease caused by genetic factors and environmental factors. A single hypothesis might not fully explain the full incidence of AD [5, 6]. Current researches indicated that the main pathological feature of AD patients was that A*β* aggregated into senile plaques and abnormal accumulation of intracellular Tau protein to form the neurofibrillary tangles (NFT) and neuronal death [7]. The current mainstream detection methods were shown in Table 1 [8–11], but each method was not perfect. Therefore, in this study, we expected to explore more suitable detection methods through bioinformatics analysis. In recent years, bioinformatics prediction and computer technology had been well applied in all aspects of cancer researches [12]. Consequently, through bioinformatics analysis, we tried to find out the cause of AD and develop a reasonable and feasible early detection program.

The main clinical manifestations of AD are memory loss and cognitive impairment. And the hippocampus (HIP) is the major functional area of the brain responsible for learning and memory. As the early reliable detection method is still poorly provided, many AD biomarkers compared the differences of microRNAs or RNA between AD and healthy elderly people, while cerebrospinal fluid (CSF) is still needed for detection. Blood collecting is more convenient than CSF, and the accuracy of biomarkers could be improved by identifying genes codifferentially expressed in HIP and blood. So we focus on the patients' blood to find genes that are consistent with the changes in HIP. To exploit the pathogenic targets and related biological processes of AD by the new method, we downloaded mRNA expression profiles GSE1297, GSE5281, GSE18309, and GSE4226 from the Gene Expression Omnibus database to obtain blood and hippocampal samples from AD patients.

## 2. Materials and Methods

### 2.1. Microarray Data

Microarray datasets GSE1297, GSE5281, GSE18309, and GSE4226 were downloaded from Gene Expression Omnibus and collected using the following platforms: GPL1211 NIA MGC, Mammalian Genome Collection, GPL570 [HG-U133_Plus_2] Affymetrix Human Genome U133 Plus 2.0 Array, and GPL96 [HG-U133A] Affymetrix Human Genome U133A Array.

### 2.2. Conversion of Raw Data

The raw data was converted to a recognizable format by GEO2R (https://www.ncbi.nlm.nih.gov/geo/geo2r/). GEO2R performs comparisons on original submitter-supplied processed data tables using the GEOquery package which parses GEO data into R data structures that can be used by other R packages and limma (Linear Models for Microarray Analysis) R package from the Bioconductor project. Each chip group is compared with AD and NC, respectively.

### 2.3. Difference Analysis

Difference analysis was performed by GEO2R analysis with* P* < 0.05 and |logFC| > 1 as cutoff values for screening differentially expressed genes (DEGs). Enrichment analysis was performed by a Cytoscape [13] plug-in ClueGO [14] with* P* < 0.05. ClueGO performed single-cluster analysis and comparison of several clusters. The ClueGO network was created with kappa statistics and reflected the relationships between the terms based on the similarity of their associated genes. Functionally grouped network with terms as nodes was linked based on their kappa score level (≥0.3). The intersection of the two sets of DEGS was obtained by VENN diagram. Enrichment results of each chip were term* P* value, corrected with Bonferroni methods.

## 3. Results

**Figure 1:**
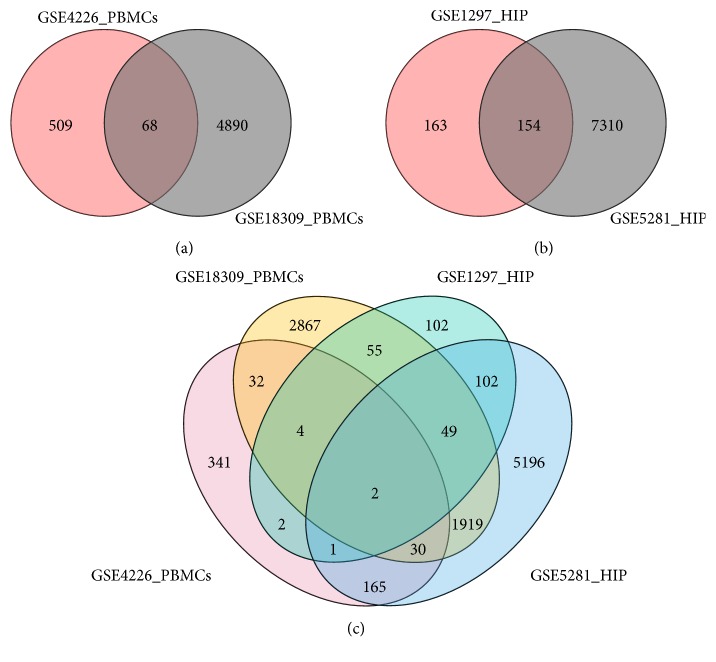
Analysis of differentially expressed genes in AD PBMCs and HIP samples by Venn. (a) Differential mRNAs expression in PBMCs of AD patients. (b) Differential mRNAs expression in HIP of AD patients. (c) Coexpression of differentially expressed mRNAs in the PBMCs and HIP of AD patients.

We compared the AD peripheral blood mononuclear cells (PBMCs) expression dataset (GEO accession GSE4226 and GSE18309) with AD brain tissue expression datasets (GEO accessions GSE1297 and GSE5281) from GEO in the present study. As shown in Table 2, for PBMCs, GSE18309 and GSE4226 included 3 and 14 samples of elderly and 6 and 14 samples of AD patients. For HIPs, GSE1297 and GSE5281 included 9 and 13 elderly samples and 22 and 10 AD samples, respectively. Gene difference analysis found that there were 4958 differential mRNAs in GSE18309, 577 differential mRNAs in GSE4226, 7464 differential mRNAs in GSE5281, and 317 differential mRNAs in GSE1297 compared with AD patients and healthy elderly. The edger differential gene analysis was performed by using the GEO language analysis tool GEO2R coming with the GEO website. Venn indicated that there were 68 differential mRNAs' expressions in both PBMCs' microarrays (Figure 1(a), Supplemental Table 1) and 154 mRNAs in the two HIP microarrays (Figure 1(b), Supplemental Table 2). In order to obtain the target differential expression gene, we further got two differentially expressed genes, RAB7A and ITGB1, in PBMCs and HIP (Figure 1(c)).

**Figure 2:**
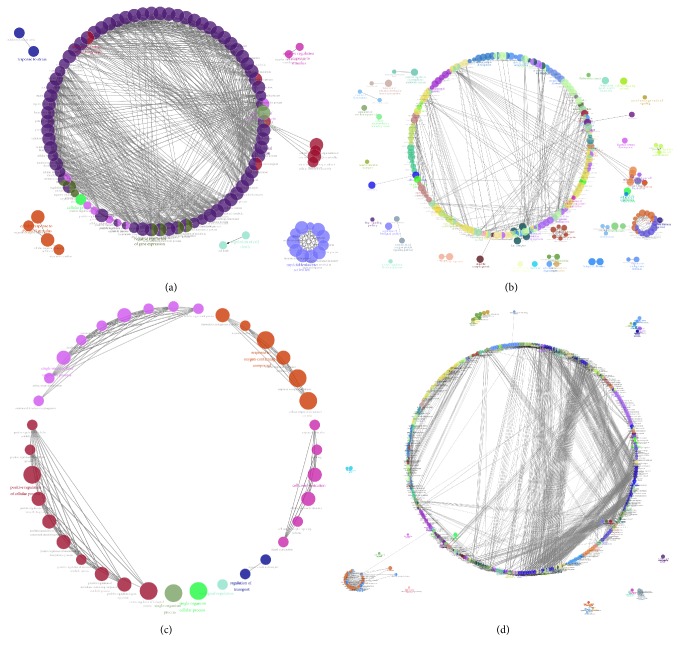
Enrichment network analysis of GSE4226 (a), GSE18309 (b), GSE1297 (c), and GSE5281 in the PBMCs or HIP of AD patients. To further capture the relationship between terms, a subset of enriched terms was selected and drawn as a network graph. Their representative enriched terms were colored by different color. The line showed the relative of spot that was connected. Functionally grouped network with terms as nodes was linked based on their kappa score level (≥0.3), where only the label of the most significant term per group is shown in color.

**Figure 3:**
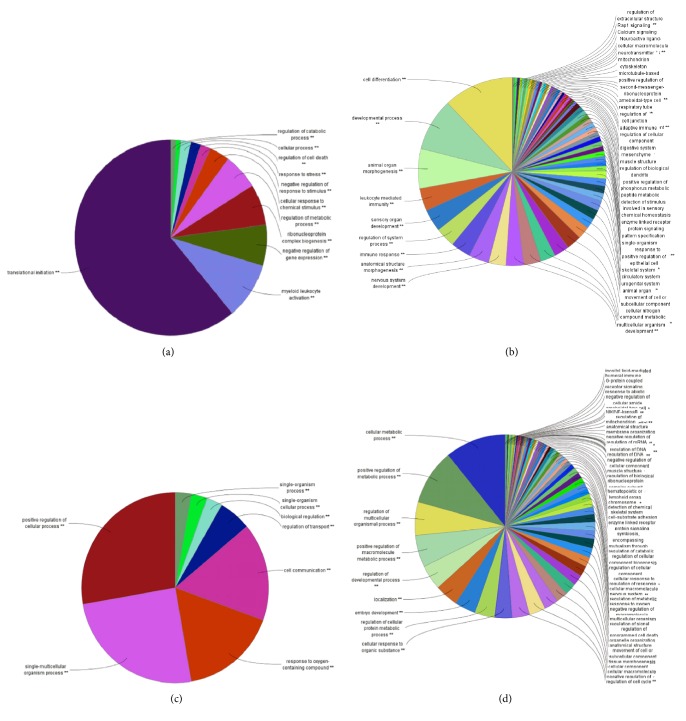
Overview chart with functional groups of each chip with term P value corrected. (a) GSE4226. (b) GSE18309. (c) GSE1297. (d) GSE5281.

Studying the signaling pathway of gene involvement could help us to understand the role of gene in AD and further understand the pathological process or cause of AD. ITGB1 was a receptor for IL1*β* and binding was essential for IL1*β* signaling [15]. RAB7A was a key regulator in endolysosomal trafficking [16], playing important roles in neurotrophin transport, lipid metabolism, microbial pathogen infection, and survival [17, 18]. These two genes were also reported as important regulators associated with AD [19–21]. In order to analyze the possible signaling pathways of RAB7A and ITGB1 in AD, we further enriched and analyzed the four chips. We enriched these genes and analyzed them through the Cytoscape plug-in ClueGo (Figure 2, details shown in Supplemental Figures 1–4). ClueGO integrates GO terms as well as KEGG/BioCarta pathways and creates a functionally organized GO/pathway term network. The network is automatically laid out using the organic layout algorithm supported by Cytoscape. ClueGo also provides the option to calculate mid P values and doubling for two-sided tests to deal with discreetness and conservatism effects as suggested (Figure 3, details shown in Supplemental Figures 5–8).

We searched for the enrichment of RAB7A and ITGB1 and select the first five results with the lowest term* P* value corrected with Bonferroni step-down. In HIP, single-organism process, positive regulation of cellular and biological process, and cell communication were enriched in GSE1297, and positive or negative regulation of metabolic process, organelle organization, and signaling regulation were enriched in GSE5281. According to these enrichment results, it was indicated that RAB7A and ITGB1 in HIP were regulatory genes response to metabolism, signaling transduction, and cellular function and communication in AD brain (Table 3, Figures 2(c) and 2(d), and Figures 3(c) and 3(d)). Furthermore, in PBMCs, symbiosis (encompassing mutualism through parasitism), viral process and life cycle, catabolic process, and cellular protein localization were enriched in GSE4226; immune response, locomotion, regulation of localization, leukocyte activation, and signaling were enriched in GSE18309 (Table 3, Figures 2(a) and 2(b), and Figures 3(a) and 3(b)). It was suggested that immune response and inflammatory changes to the microorganism or stimuli were the main changes in AD PBMCs.

As far as the two genes are concerned, the involving functions in PBMCs were not so similar to those in HIP. However, we found that they had close relationship with each other. Activation of immune response releases many proinflammatory cytokines. Long-term activation of the immune system in AD exacerbated neuroinflammation and contributes to neurodegeneration [22]. Recruitment of peripheral macrophages into the brain led to deterioration of AD pathology, which might aggravate the effects of persistent inflammation and AD pathology [23]. Also, neurodegeneration further stimulated the immune system to become a malignant cycle and caused systemic effects [24]. Therefore, it meant that the changes of RAB7A and ITGB1 in PBMCs could be premonitory signs of AD which would lead to some consequent biological changes in HIP and finally to cognitive problems.

## 4. Discussion

AD was an irreversible progressive neurodegenerative disease that can only be controlled by early intervention for early prevention and treatment. To date, there are mainly MMSE, medical imaging, or biomarkers to detect AD, but the early prediction results were not satisfactory, suggesting that new early biomarkers needed to be obtained, and effective and efficient detection schemes should be developed.

At present, bioinformatics prediction and computer technology had been well applied in all aspects of biomedicine research [25]. However, there are not many bioinformatics analyses in Alzheimer's disease, and the current research on Alzheimer's disease was mainly concentrated in single site, which lacks multiple sites for joint research. Chip analysis and screening allow researchers to obtain many differential genes. The expressed data provided a novel approach to study the detection and targeted therapy of AD or to exploit new biomarker.

By comparing the hippocampus and peripheral blood of AD patients with normal elderly group, 154 differentially expressed genes in hippocampus and 68 differentially expressed genes in peripheral blood were obtained. We found that ITGB1 and RAB7A were differentially expressed in HIP and PBMCs of AD patients by Venn diagram compared with healthy elderly people. RAB7A was involved in tau secretion in the brain and played an important role in the transport of neurotrophic factors in animal and cell experiments [21, 26, 27]. Besides, RAB7A plays a key role in neurotrophin transport and lipid metabolism in neuronal axons [17]. At the same time, RAB7A also plays an important role in the infection and survival of microbial pathogens [18]. The other screened gene was ITGB1, which is a receptor for IL1**β** and binding is essential for IL1**β** signaling [15]. As we know, IL1**β** plays a key role in the occurrence and development of AD [28], so ITGB1 also contributed to AD progression. It was reported that ITGB1 interacted with APP to regulate axons outward growth and adhesion and affects cell migration, which was supported by many literatures [21, 26, 27]. Moreover, ITGB1 plays an important role in the regulation of immune response [29], consistent with our results. Even though their biological function enrichment in PBMCs was not similar to those in HIP, there was a close relationship between the inflammatory and immune changes in peripheral blood and the signaling regulation in HIP. The immune system secretes proinflammatory cytokines, leading to the emergence of responses [30]. Inflammation plays an important role in the pathogenesis of AD. Current studies have shown that there are strong inflammatory reactions mediated by microglia and astrocytes in AD patients' brain [31]. An accompanying change of metabolism, signaling, and biological process in HIP followed after the immune and inflammatory changes in PBMCs.

According to our screening results, ITGB1 and RAB7A were potential biomarkers for the prediction and diagnosis of AD. Therefore, this meant that the patients could be diagnosed by detecting peripheral blood, without brain biopsy or cerebrospinal fluid detection. However, the patient's blood samples can be detected by PCR to determine the condition; the threshold for the diagnosis of AD needs to be determined by experiments. In addition to detecting the expression of ITGB1 and RAB7A for AD screening, we recommended that the routine blood of the elderly was tested for 2-3 years at a frequency of 6 months or 1 year. If long-term immune system abnormalities are found in the elderly, the patients need to be treated or intervened as earlier as possible. However, the cut-off value should be set in normal physiological range of normal healthy population. This would be conducive to hospital implementation and diagnosis. Although inflammation did not necessarily lead to AD, AD patients usually have a long-term inflammatory response.

We believed that inflammation is a signal of early pathological changes in AD. Severe inflammatory reactions in middle-aged people had been reported to increase the risk of AD [32]. Therefore, we speculated that the risk of AD in elderly patients with long-term immune system abnormalities is much higher than that in normal elderly people. The treatment or intervention of inflammatory reaction could effectively delay or delay the occurrence and development of AD. On the other hand, elderly patients with long-term immune system abnormalities also needed to be treated. For potential high-risk patients, hospital should treat or intervene in the inflammation of patients before the clinical manifestation emerging in order to achieve the effects of treatment and prevention of early AD.

## 5. Conclusions

ITGB1 and RAB7A mRNA expressions were both changed in hippocampus and PBMCs, highly suggested being used as an AD biomarker with AD. Also, according to the results of this analysis, it is indicated that we can test the blood routine of the elderly for 2-3 years at a frequency of 6 months or one year. When a patient continuously detects the inflammatory manifestations, they are indicated as a potentially high-risk AD patient for AD prevention.

## Figures and Tables

**Table 1:** Current mainstream detection methods of AD.

Detection method	Principle	Advantage	Disadvantage
MMSE	The most common scale of intelligence impairment at present. Illiteracy ≤ 17 points, primary school degree ≤ 20 points, middle school degree ≤ 22 points, college degree ≤ 23 points, indicates the impairment of cognitive function.	Low cost, concise content, less time for measurement, and easy to be accepted by the elderly	Only screening the patients with moderate-to-severe symptoms

Structural imaging: head CT (thin scan) and MRI (coronal) scanning	Diagnostic imaging techniques show significant atrophy of the cerebral cortex, especially in the hippocampus and medial temporal lobe, supporting the clinical diagnosis of AD.	Used to exclude other underlying diseases and to identify specific imaging findings of AD	Convenience not as good as MMSE, expensive cost, hospital equipment support, low penetration rate

18FDG-PET	It shows the reduction of glucose metabolism in the dome and upper/posterior temporal region, posterior cingulate cortex, and anterior cranial lobe, revealing specific abnormal changes in AD. In the late period of AD, the frontal lobe metabolism was reduced.	Improving the reliability of dementia diagnosis and being suitable for the differential diagnosis of AD and other dementia	Convenience not as good as MMSE, expensive cost, specific equipment support needed, penetration rate

EEG	The EEG of AD is characterized by a decrease in alpha wave, an increase in theta wave, and a decrease in the average frequency.	Used for the differential diagnosis of AD, providing early evidence of prion disease or other brain diseases	33% of patients with early AD are normal without change in EEG and low penetration rate

Biological markers	Dynamically observing the progression of the disease by using proteins or miRNAs in body fluids such as peripheral blood, cerebrospinal fluid, and urine biology as markers to increase the specificity of the diagnosis.	Noninvasive, simple and inexpensive, and of low price	Low repeatability, problems with accuracy and specificity, lack of standards

**Table 2:** GEO microarray datasets.

Chip serial number	Elderly sample	AD sample	Resource	Platform
GSE1297	9	22	Hippocampus	GPL1211 NIA MGC, Mammalian Genome Collection

GSE5281	13	10	Hippocampus	GPL1211 NIA MGC, Mammalian Genome Collection

GSE18309	3	6	PBMCs	GPL570 [HG-U133_Plus_2] Affymetrix Human Genome U133 Plus 2.0 Array

GSE4226	14	14	PBMCs	GPL96 [HG-U133A] Affymetrix Human Genome U133A Array

**Table 3:** TOP lowest term P value corrected with Bonferroni step-down of RAB7A and ITGB1.

CHIP	GOID	GO Term	Number of Genes	Associated genes (%)	Term P value	Term P value corrected with Bonferroni step-down
GSE4226	GO:0044403	Symbiosis, encompassing mutualism through parasitism	113	10.53122	5.98E-33	1.23E-30
	GO:0016032	Viral process	109	10.62378	4.57E-32	9.32E-30
	GO:0019058	Viral life cycle	74	15.16394	1.85E-31	3.76E-29
	GO:0009056	Catabolic process	146	5.968929	4.54E-17	8.63E-15
	GO:0034613	Cellular protein localization	121	6.547619	6.10E-17	1.15E-14

GSE18309	GO:0006955	Immune response	314	14.47672	1.25E-08	8.10E-06
	GO:0040011	Locomotion	443	24.7072	8.99E-11	6.07E-08
	GO:0032879	Regulation of localization	601	22.68781	8.00E-08	5.08E-05
	GO:0045321	Leukocyte activation	178	13.69231	3.53E-07	2.20E-04
	GO:0023052	Signaling	1386	20.76716	4.25E-07	2.64E-04

GSE1297	GO:0044699	Single-organism process	248	1.814856	1.08E-07	1.16E-05
	GO:0044763	Single-organism cellular process	213	1.933551	1.77E-07	1.86E-05
	GO:0048522	Positive regulation of cellular process	117	2.322811	7.89E-07	8.13E-05
	GO:0048518	Positive regulation of biological process	125	2.214349	3.47E-06	3.51E-04
	GO:0007154	Cell communication	139	2.078039	2.60E-05	0.002369

GSE5281	GO:0010604	Positive regulation of macromolecule metabolic process	1215	39.69291	4.13E-28	4.28E-25
	GO:0009893	Positive regulation of metabolic process	1299	39.23286	8.40E-28	8.72E-25
	GO:0006996	Organelle organization	1460	38.04065	1.36E-24	1.40E-21
	GO:0048523	Negative regulation of cellular process	1681	36.92882	6.16E-22	6.32E-19
	GO:0009966	Regulation of signal transduction	1131	38.67989	3.85E-21	3.94E-18

## Data Availability

The data used to support the findings of this study are available from the corresponding author upon request.

## Supplementary Materials

Supplementary Materials Supplemental Table 1 is the list of 68 differential mRNAs' expressions in both PBMCs' microarrays. Supplemental Table 2 is the list of 154 mRNAs in the two HIP microarrays. Supplemental Figures 1–4 correspond to Figures 2(a)–2(d). Supplemental Figures 5–8 correspond to Figures 3(a)–3(d).
