# Supplementary figures and images for: Differential Expression of mRNAs in the Brain Tissues of Patients with Alzheimer's Disease Based on GEO Expression Profile and Its Clinical Significance
Source: Biomed Res Int. 2019 Feb 26;2019:8179145. doi: 10.1155/2019/8179145 (PMC6413412; doi:10.1155/2019/8179145)

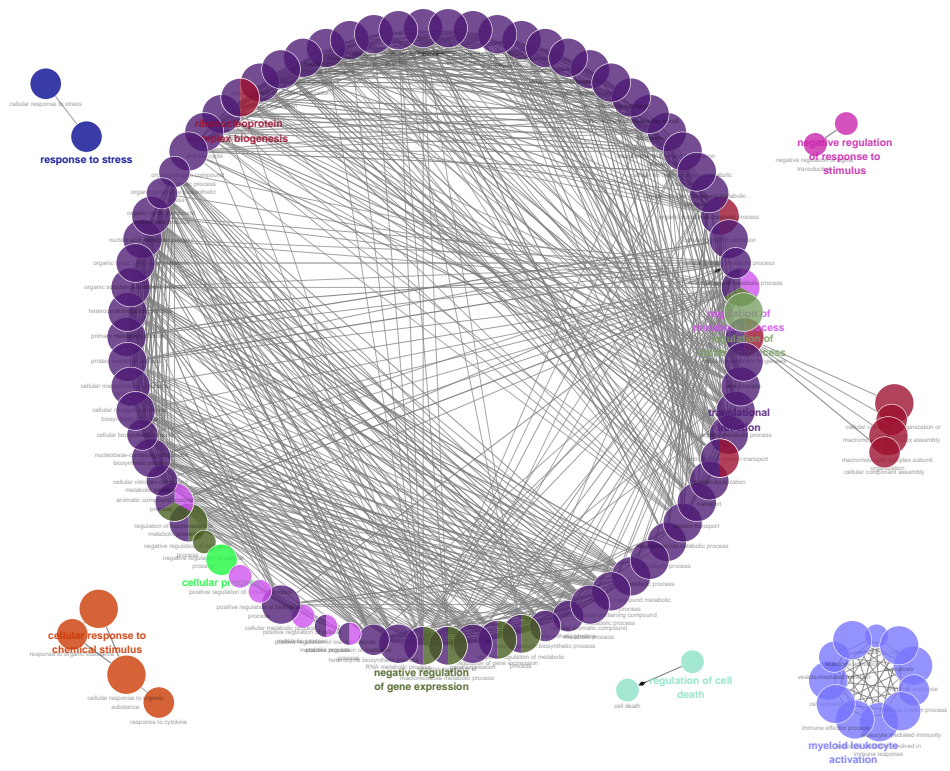

Supplement: Supplementary Materials — Supplemental Table 1 is the list of 68 differential mRNAs' expressions in both PBMCs' microarrays. Supplemental Table 2 is the list of 154 mRNAs in the two HIP microarrays. Supplemental Figures 1–4 correspond to Figures 2(a)–2(d). Supplemental Figures 5–8 correspond to Figures 3(a)–3(d). [file 8179145.f1.zip › 1.pdf]

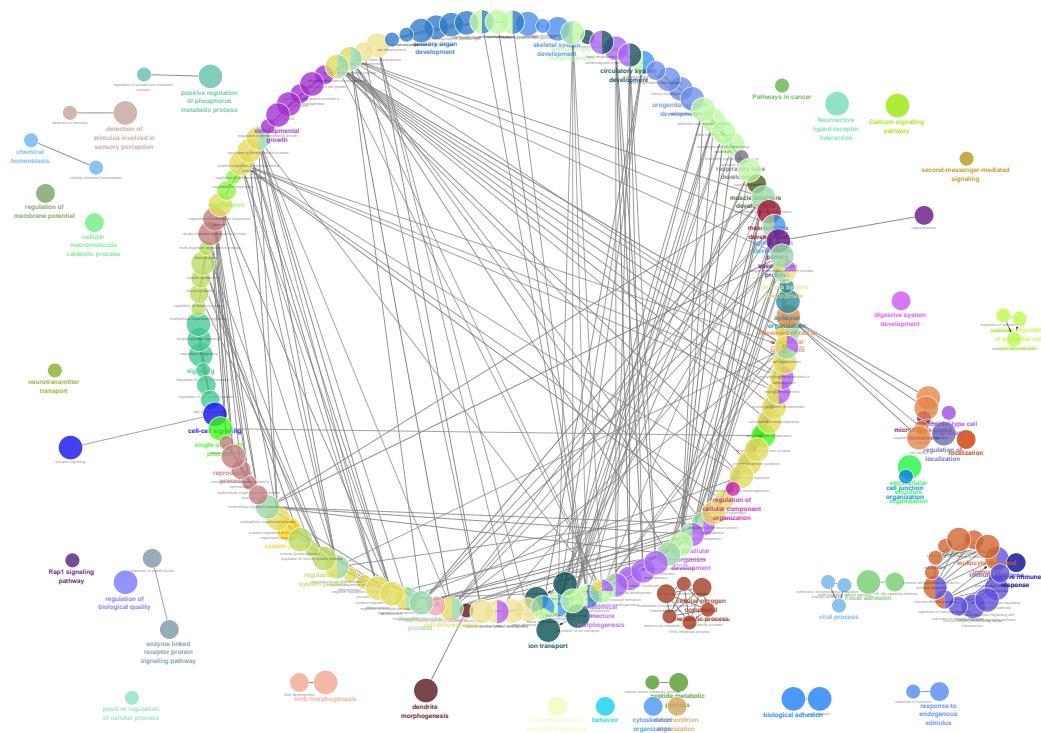

Supplement: Supplementary Materials — Supplemental Table 1 is the list of 68 differential mRNAs' expressions in both PBMCs' microarrays. Supplemental Table 2 is the list of 154 mRNAs in the two HIP microarrays. Supplemental Figures 1–4 correspond to Figures 2(a)–2(d). Supplemental Figures 5–8 correspond to Figures 3(a)–3(d). [file 8179145.f1.zip › 2.pdf]

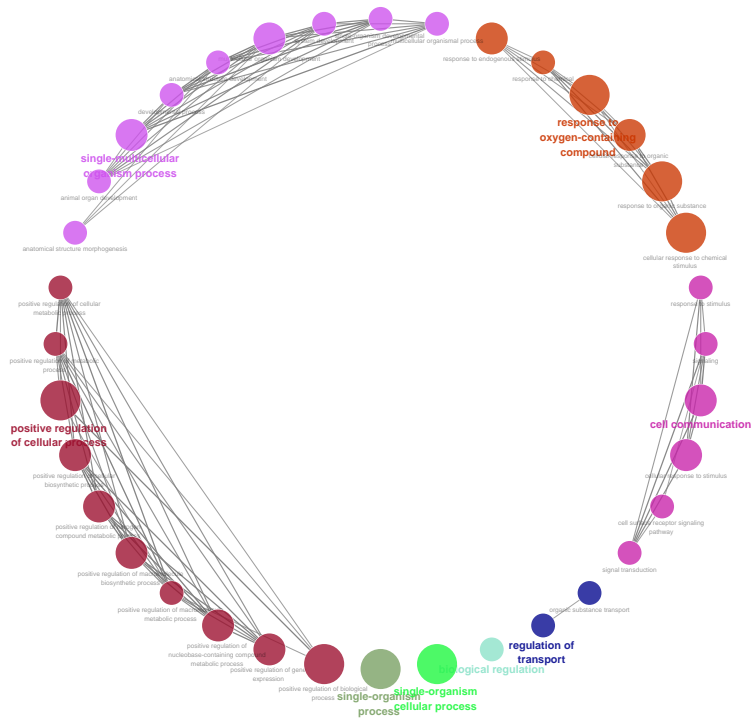

Supplement: Supplementary Materials — Supplemental Table 1 is the list of 68 differential mRNAs' expressions in both PBMCs' microarrays. Supplemental Table 2 is the list of 154 mRNAs in the two HIP microarrays. Supplemental Figures 1–4 correspond to Figures 2(a)–2(d). Supplemental Figures 5–8 correspond to Figures 3(a)–3(d). [file 8179145.f1.zip › 3.pdf]

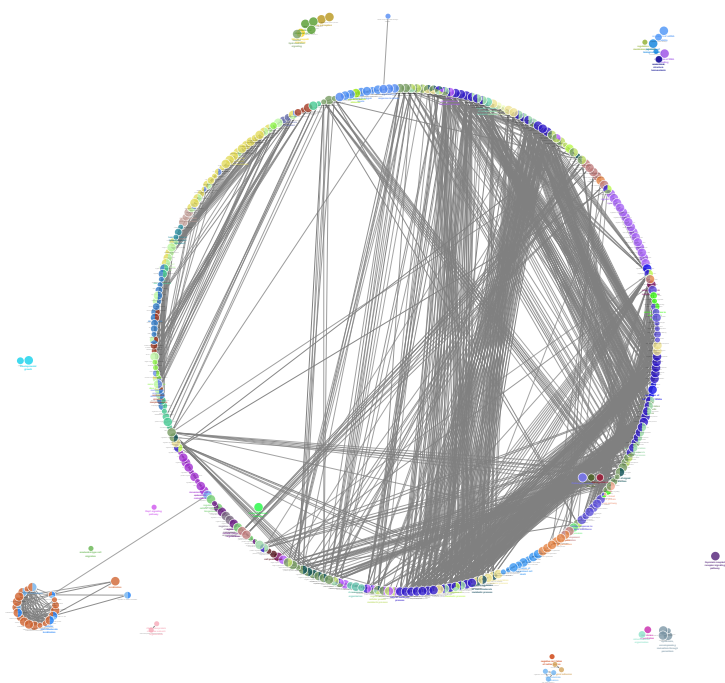

Supplement: Supplementary Materials — Supplemental Table 1 is the list of 68 differential mRNAs' expressions in both PBMCs' microarrays. Supplemental Table 2 is the list of 154 mRNAs in the two HIP microarrays. Supplemental Figures 1–4 correspond to Figures 2(a)–2(d). Supplemental Figures 5–8 correspond to Figures 3(a)–3(d). [file 8179145.f1.zip › 4.pdf]

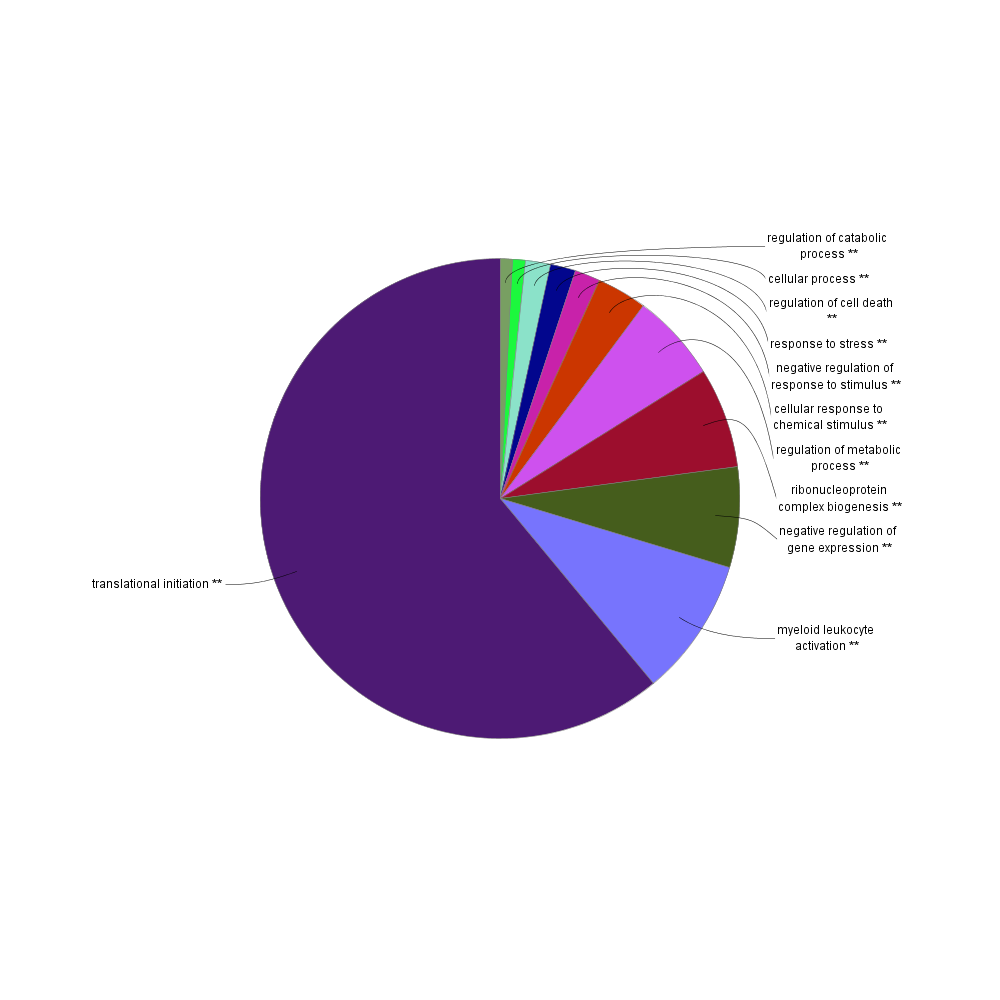

Supplement: Supplementary Materials — Supplemental Table 1 is the list of 68 differential mRNAs' expressions in both PBMCs' microarrays. Supplemental Table 2 is the list of 154 mRNAs in the two HIP microarrays. Supplemental Figures 1–4 correspond to Figures 2(a)–2(d). Supplemental Figures 5–8 correspond to Figures 3(a)–3(d). [file 8179145.f1.zip › 5.png]

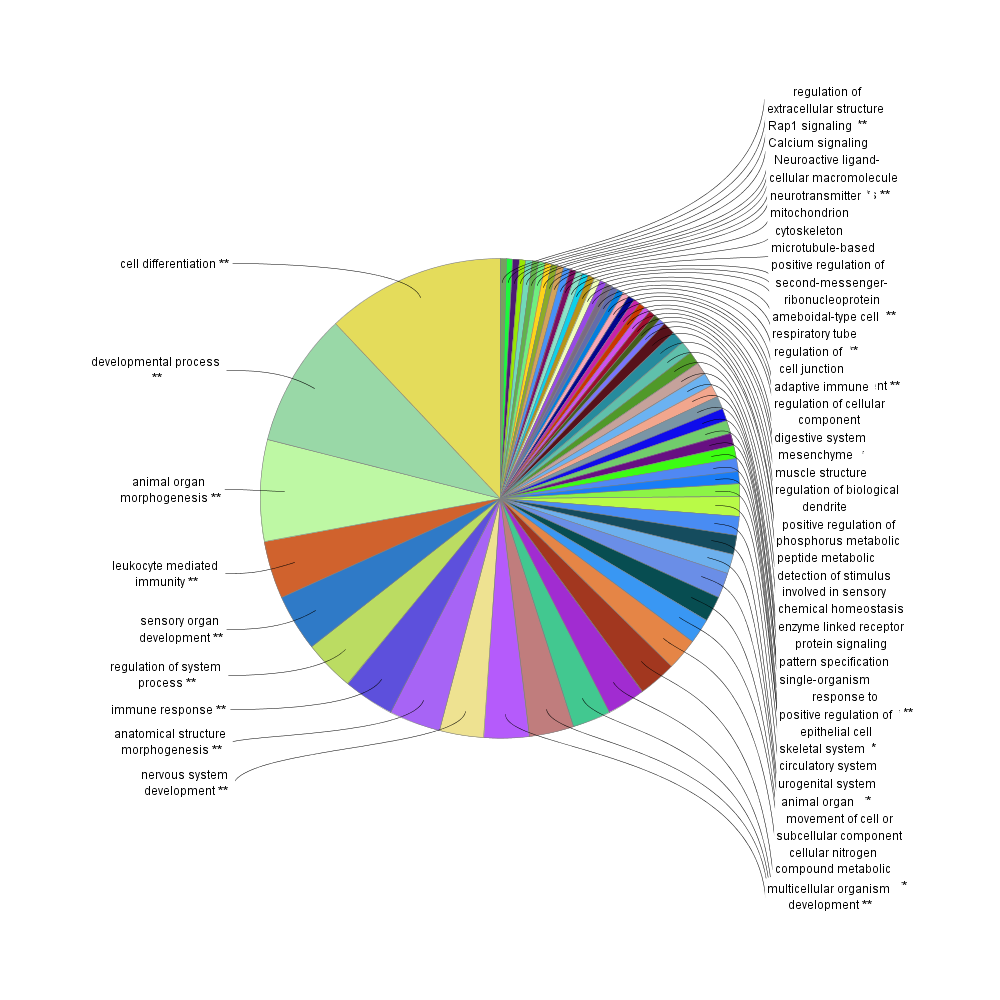

Supplement: Supplementary Materials — Supplemental Table 1 is the list of 68 differential mRNAs' expressions in both PBMCs' microarrays. Supplemental Table 2 is the list of 154 mRNAs in the two HIP microarrays. Supplemental Figures 1–4 correspond to Figures 2(a)–2(d). Supplemental Figures 5–8 correspond to Figures 3(a)–3(d). [file 8179145.f1.zip › 6.png]

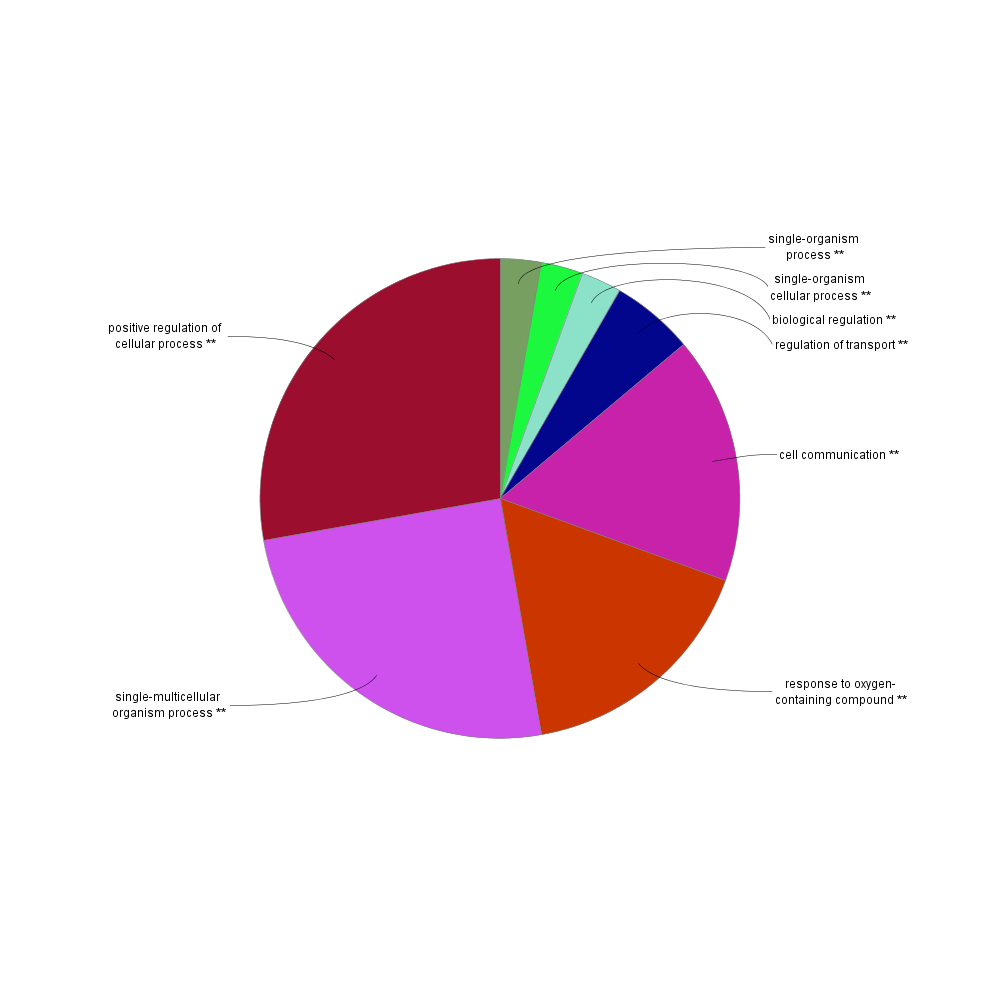

Supplement: Supplementary Materials — Supplemental Table 1 is the list of 68 differential mRNAs' expressions in both PBMCs' microarrays. Supplemental Table 2 is the list of 154 mRNAs in the two HIP microarrays. Supplemental Figures 1–4 correspond to Figures 2(a)–2(d). Supplemental Figures 5–8 correspond to Figures 3(a)–3(d). [file 8179145.f1.zip › 7.png]

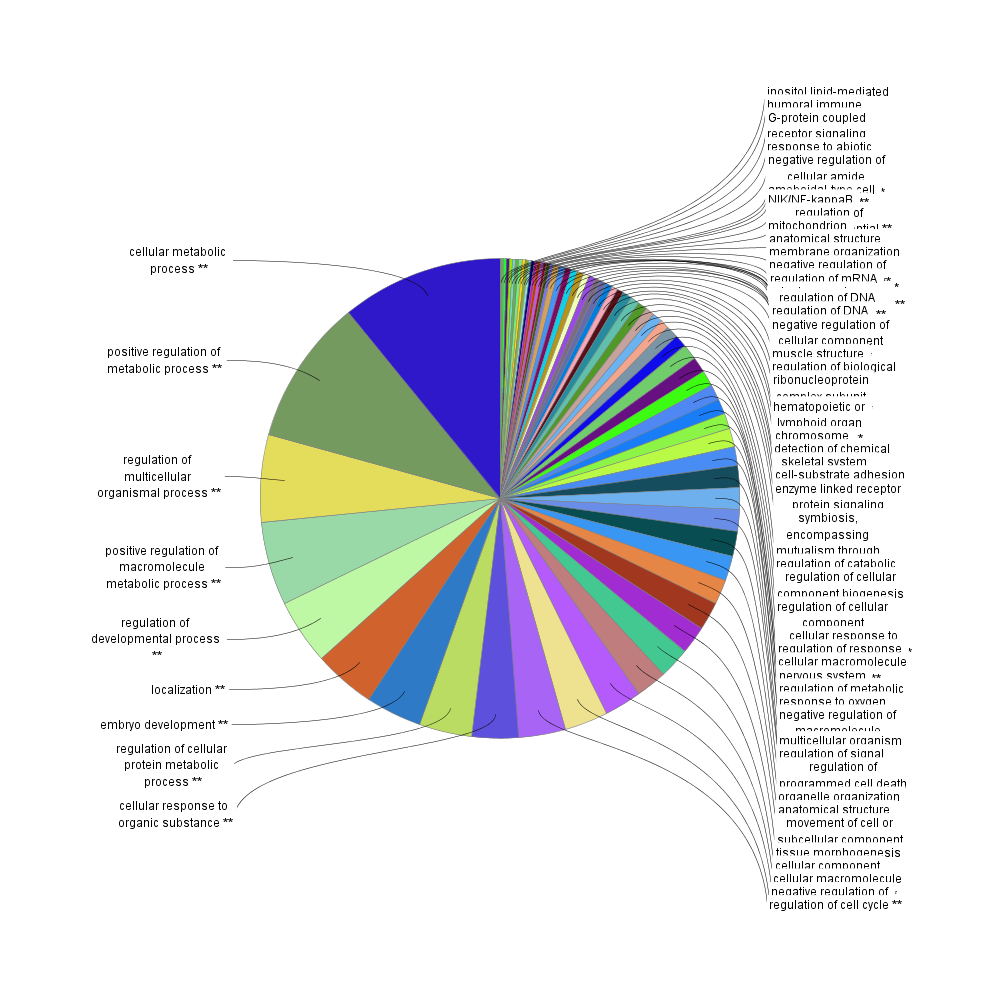

Supplement: Supplementary Materials — Supplemental Table 1 is the list of 68 differential mRNAs' expressions in both PBMCs' microarrays. Supplemental Table 2 is the list of 154 mRNAs in the two HIP microarrays. Supplemental Figures 1–4 correspond to Figures 2(a)–2(d). Supplemental Figures 5–8 correspond to Figures 3(a)–3(d). [file 8179145.f1.zip › 8.png]
